# COVID‐19‐associated facial cutaneous mucormycosis superinfection: A potentially life‐threatening disease

**DOI:** 10.1002/ccr3.6103

**Published:** 2022-07-22

**Authors:** Zahra Zareshahrabadi, Amir Emami, Keyvan Pakshir, Amir Roudgari, Behzad Ghaffari, Tahere Rezaei, Golsa Shekarkhar, Kamiar Zomorodian

**Affiliations:** ^1^ Basic Sciences in Infectious Diseases Research Center Shiraz University of Medical Sciences Shiraz Iran; ^2^ Microbiology Department, Burn & Wound Healing Research Center Shiraz University of Medical Sciences Shiraz Iran; ^3^ Department of Parasitology and Mycology, School of Medicine Shiraz University of Medical Sciences Shiraz Iran; ^4^ Trauma Research Center, Shahid Rajaee (Emtiaz) Trauma Hospital Shiraz University of Medical Sciences Shiraz Iran; ^5^ Vice Chancellor for Treatment Shiraz University of Medical Sciences Shiraz Iran; ^6^ Department of Pathology, School of Medicine Shiraz University of Medical Sciences Shiraz Iran

**Keywords:** coronavirus infections, facial skin, fatal infection, mucormycosis

## Abstract

A 49‐year‐old male was involved in an accident and an abdominal computer tomographic examination revealed papillary renal cell carcinoma of the right kidney. During hospitalization, the patient was infected with COVID‐19. In the following COVID‐19 treatment, a black dot developed on the right side of the head and face. Antifungal therapy and surgical debridement were initiated and gradual improvement was observed.

## Abstract 2

COVID‐19 infection is spreading rapidly and mucormycosis is an invasive fungal infection which causes a high rate of mortality. Early diagnosis and prompt treatment can result in better outcome of the patient.

## 1 INTRODUCTION

Corona virus disease 2019 (COVID‐19) is a contagious viral respiratory disease that is still a major global cause of mortality. The disease is characterized by common signs and symptoms such as fever, fatigue, dry cough, and shortness of breath, and it can also cause acute respiratory distress syndrome (ARDS) in severe cases.^1^ As a matter of fact utilization of corticosteroids in COVID‐19 hospitalized patients to modulate inflammation‐mediated lung injury and lower mortality rates may expose patients to opportunistic bacterial and fungal infections which in turn may lead to death.^2^

Currently, mucormycosis, an invasive fungal infection associated with COVID‐19 infection, can cause widespread dissemination during treatment or after hospital discharge. Mucormycosis is a rare, angioinvasive fungal infection that rapidly progresses with significant morbidity and mortality.^3^ Cutaneous mucormycosis due to angioinvasion has the potential for rapid death if treatment is not proper and quick.^4^, ^5^

Previous studies and literature have shown that mucormycosis is a highly aggressive disease, highlighting the necessity of early diagnosis and treatment initiation. Systemic antifungal medication, primarily intravenous amphotericin B (AMB), and vigorous surgical debridement, are the cornerstones of mucormycosis treatment.^4^, ^6^

The association of these two critical infectious diseases is challenging for all the people of the World. In this case, we presented a severe car accident case where doctors incidentally identified type 2 renal papillary cell cancer, COVID‐19 infection, and subsequent co‐infection with facial cutaneous mucormycosis.

## 2 CASE HISTORY

### 2.1 General presentation

A 49‐year‐old male was involved in a car accident, emergency personnel discovered him facing downward on a gravel road. The injured man was promptly sent to the level I trauma center of Rajaei hospital, where his multiple head, face wounds were sutured and treated. The patient underwent surgery due to a pelvic bone extremity fracture. Following a car accident‐related hospitalization, standard medical examination and diagnostics revealed an apparent anomaly in the patient's abdomen. Abdominal CT revealed spherical soft tissue masses in the right kidney's patient. The mass was fully removed during a nephrectomy operation, and sent for histopathological examination.

### 2.2 Laboratory investigations

According to the histological morphology results, the maximum diameter of the removed mass was 12 × 6 × 5 cm with >60% necrosis, which was pathologically diagnosed as type 2 papillary renal cell carcinoma (PRCC) in stage I, according to World Health Organization (WHO)/International Society of Urological Pathology (ISUP) classification.^7^ No lymphovascular and regional lymph node invasions were identified and the margins could not be assessed. During post‐surgery, hospitalization, the patient complained of fever, dyspnea, cough, fatigue, and shortness of breath as the most common symptoms of coronavirus infection, during the spread of COVID‐19 pneumonia. Additionally, the case had no immune system disorder history and HIV, HBs antigen (HBsAg), and HCV Ab serology tests were all negative.

**FIGURE 1:**
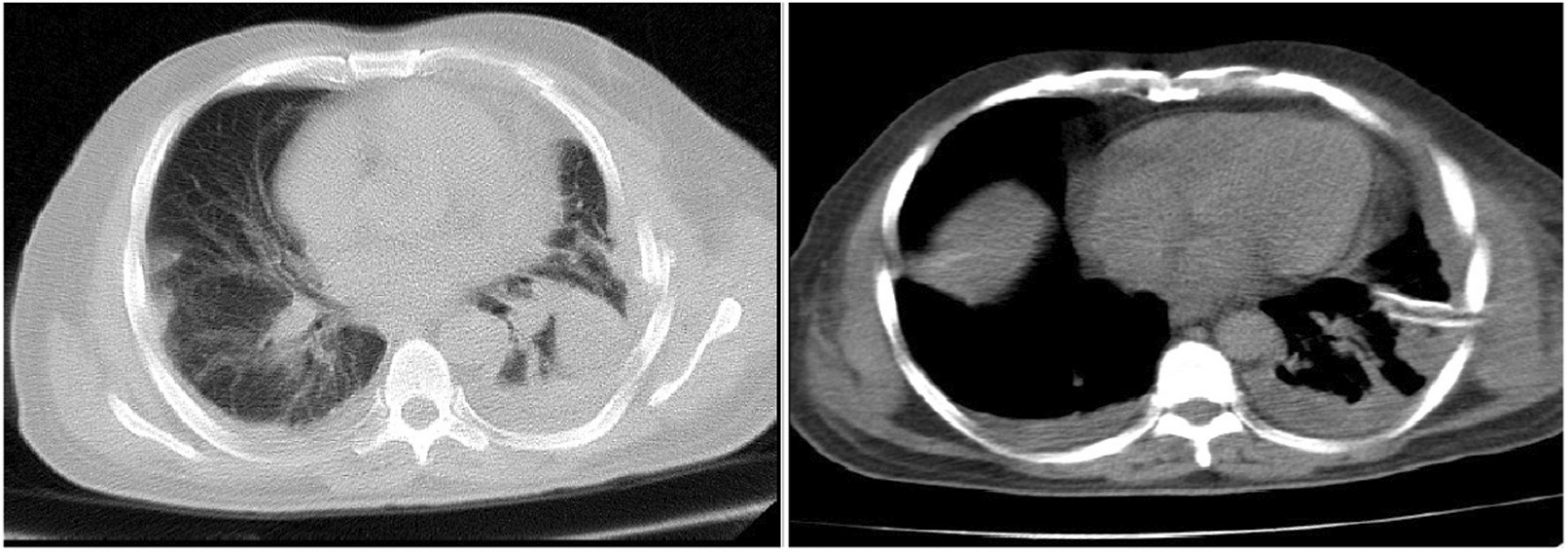
Axial views of the chest HRCT scan illuminating peripheral bilateral pleural effusion and ground glass opacities for COVID‐19

A routine full blood count revealed a revealed Hb of 10.2 mg/dl, WBC: 3.4 × 10^9^/L with 60% lymphocytes, CRP and ESR were both elevated and BUN: 17 mg/dl, Cr: 1.1 mg/dl, also glucose level increased slowly from 100 to 168 mg/dl. In addition, a negative COVID‐polymerase chain reaction (PCR) from a nasopharyngeal swab was reported. Moreover, his high‐resolution chest computed tomography (HRCT) demonstrated massive bilateral pleural effusion and suspicious ground glass opacities for COVID‐19, which is located on the left side (Figure 1).

### 2.3 Management of treatment

Because of acute respiratory failure, a chest tube was placed in the chest cavity of the patient to remove the pleural effusion fluid. Clinical evidence lymphopenia, low oxygen saturation level, high level of CRP and ESR suggested COVID‐19 lung involvement. Thus, COVID‐19 treatment was initiated immediately.

The patient was admitted to the intensive care unit (ICU) and received 4 doses of intravenous dexamethasone (4 mg twice daily), interleukin antagonists, broad‐spectrum antibiotics, and remdesivir 200 mg on day 1, followed by 100 mg daily for four days. In addition, the patient received supplemental oxygen for symptom relief. In this case, the patient was being treated for a COVID‐19 infection. Eight days later; a small red dot emerged at the patient's head as a result of the accident injuries. The lesions gradually became swollen and finally necrotic with black spots. Physical examination indicated an irregular large area of red plaques, nodules, and extensive necrotic ulcers on the right side of the frontal, parietal, and temporal of his head and right zygomatic face region, as well as a black crust on the surface and edge lesions. The right eye did not open, because of edema in the surrounding area of the eye and drooping eyelids.

**FIGURE 2:**
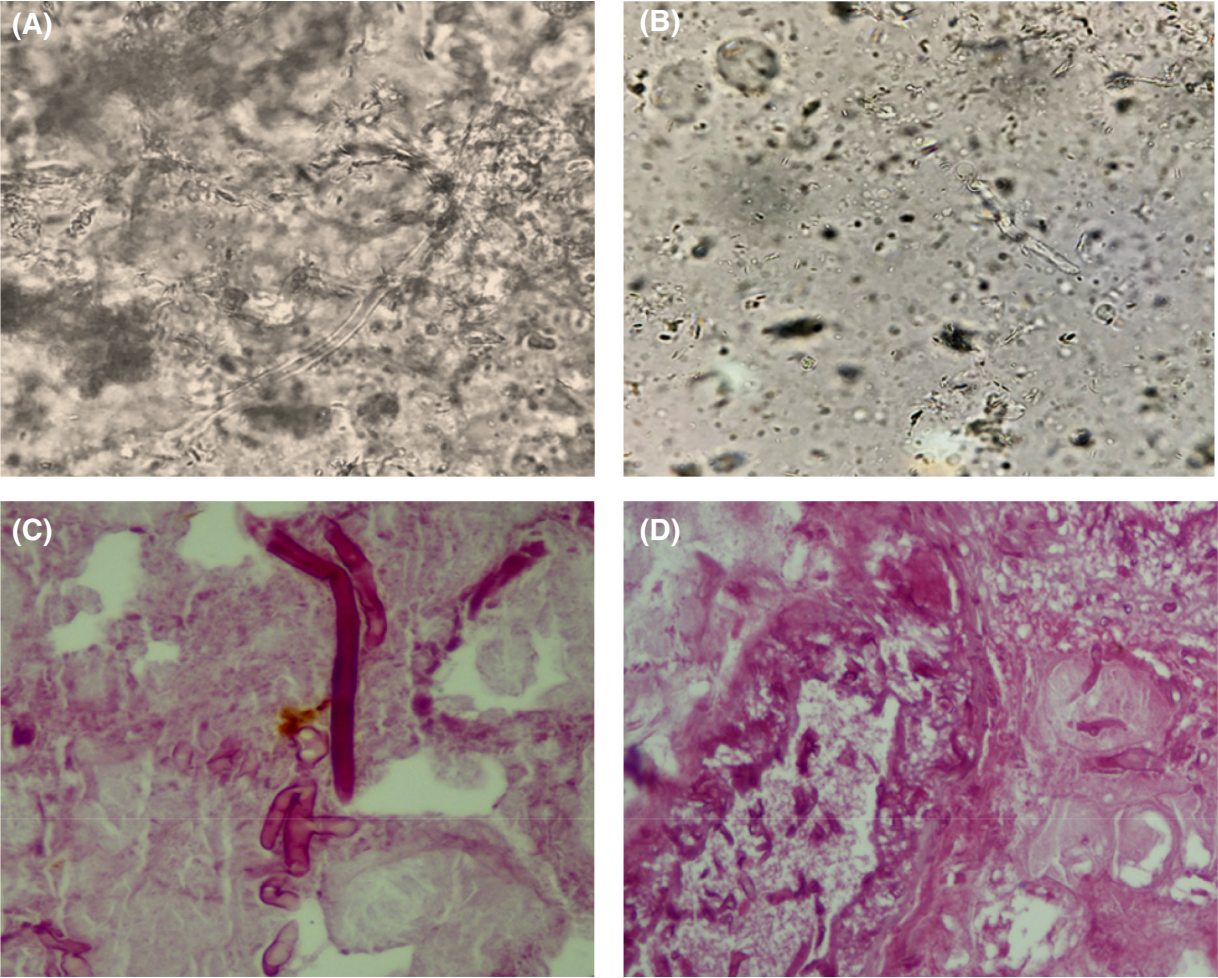
KOH wet mount examination of damaged facial tissue presenting aseptate hyphae (20% KOH, ×400) (A and B). Histopathological examination showing broad and transparent hyphae with right‐angle branching structures (H&E staining) (C and D).

Biopsies were performed, and samples were sent for mycological and pathological testing. The wet preparation of the sample with potassium hydroxide (KOH) revealed broad, non‐septate, branching hyphae typical of mucormycetes (Figure 2A,B). The culture yielded negative results, most likely as a result of pretreatment. The original tissue cultures, which included sputum, blood, and a bacterial culture of the lesion, were all negative for pathogen identification.

Hematoxylin and eosin (H&E) staining of the tissue biopsy revealed many thick‐walled, ribbon‐shaped, and nonseptate hyphae with right angle branching in the background of necrotic tissue as a pathological hallmark of mucormycosis (Figure 2C,D). Inflammatory cells were also observed. Subsequently, the patient was diagnosed with cutaneous mucormycosis of the head and face by tissue biopsy.

The patient underwent intravenous and oral antifungal therapy. Initially, antifungal treatment options consisted of intravenous liposomal amphotericin B (AMB) (5 mg/kg/day) as first‐line therapy when the patient's clinical conditions were controlled and improved. Besides, wide surgical debridement of the damaged and diseased necrotic/dead tissue was performed in the operating room.

It is noteworthy that the CT of the paranasal sinuses confirmed that all sinuses were not involved by *Mucor*; he had a mucosal thickening in the maxillary sinuses, and also brain MRI and paranasal sinus CT scan were normal without any evidence of abnormalities. However, palate necrosis, ptosis, and proptosis were not noted. After 15 days of antifungal therapy, liposomal AMB therapy was stopped and the patient with an oral itraconazole (ITR) (200 mg/day) was discharged and continued to show no evidence of mucormycosis.

**FIGURE 3:**
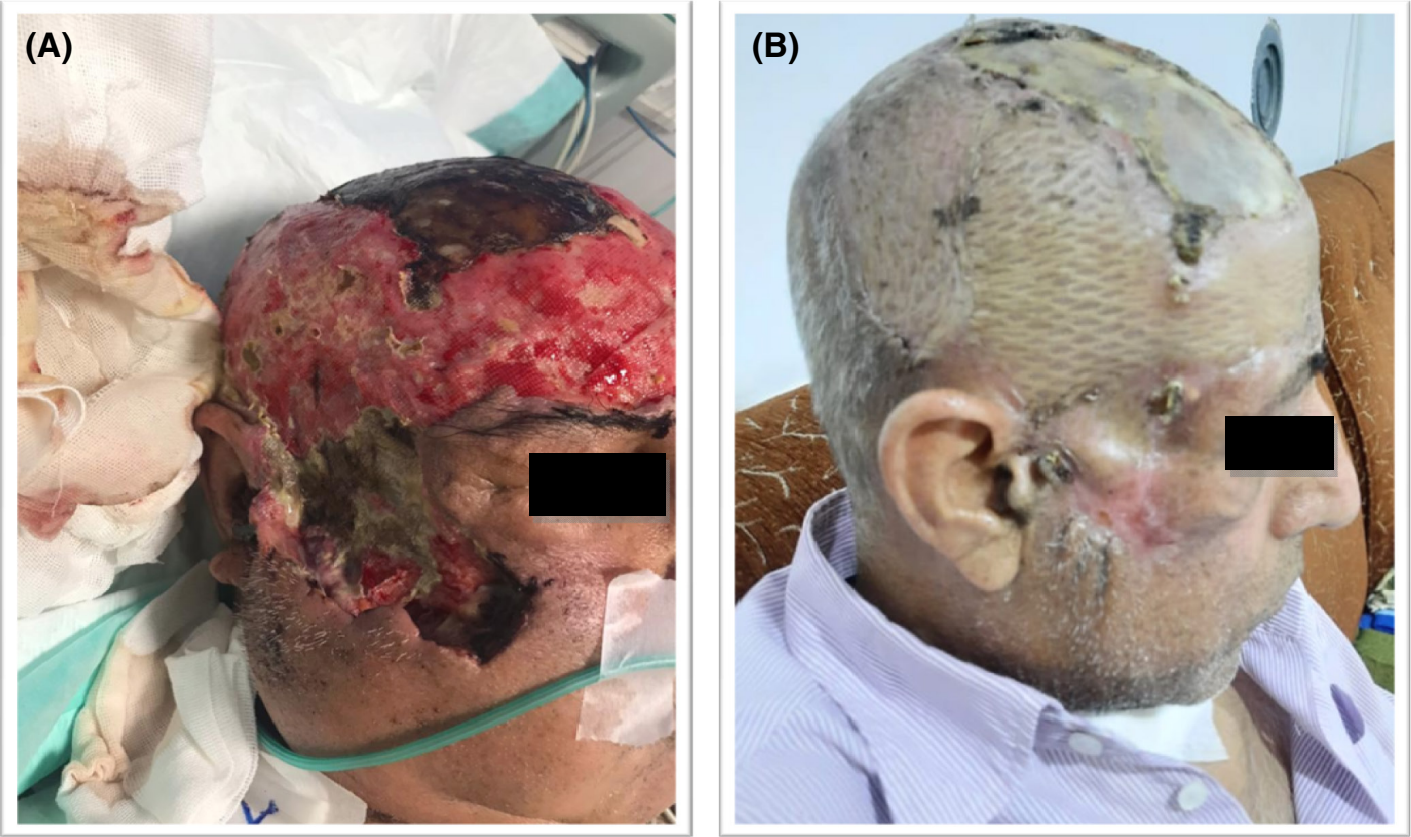
Views of facial cutaneous mucormycosis case with large area of red plaques, nodules, and necrotic tissues with black crust on the wound edge before starting treatment, extensive necrosis of scalp with erythrema and fluctuation (before reconstruction) (A). Clear healing of facial skin lesions after 4‐month treatment with AMB, ITR and plastic surgery (B).

After 85 days of hospitalization, the patient was discharged in good condition with fresh granulomatous and scar formation of the skin lesions after recovery (Figure 3B). In this case, during the follow‐up period, no recurrence and side effects of antifungal drug usage were found. Since then, appropriate treatment and recovery of swelling and erythema around the involved tissue, as well as a facial transplant, have been performed. At present, the patient is still under regular follow‐up and examination.

## 3 DISCUSSION

Secondary infections with mucormycosis as a post COVID treatment are well documented in critically ill patients with COVID‐19. On March 11th, 2020, the World Health Organization (WHO) announced it as a global public health emergency concern.^8^

Mucormycosis is a fungal emerging infection caused by Mucorales. Three genera in this family can cause clinical infection: *Rhizopus*, *Absidia*, and *Mucor*.^9^, ^10^ The prevalence of mucormycosis in the general population is higher in Europe than in Asia, as they reported 34% in Europe, followed by Asia (31%), America (28%), and Africa (3%). In general, these fungi are opportunistic pathogens with risk factors such as diabetes, malignancy, HIV infection, organ transplantation, and open wounds following trauma. According to past studies, the mortality rate in mucormycosis patients is high. For example, it ranges from 56.5% organ transplantation^11^ to 78.6% in patients with neutropenia.^12^

During the second wave of the COVID‐19 global pandemic, a large number of mucormycosis cases were observed in COVID‐19 patients, and this trend has recently increased and continues.^13^ We found a comprehensive review of literature reporting COVID‐19 associated with mucormycosis cases. Muthu et al. reported a systematic review of COVID‐19‐associated mucormycosis until June 21st, 2021 and included individual patient details of 275 cases, of which 233 were reported from India and 42 from the rest of the world.^14^, ^15^, ^16^ In this case, predisposing factors which facilitated the germination of Mucoral spores in the face and head include prolonged hospitalization, mechanical ventilation and taking high doses of systemic corticosteroids, which increased the case susceptibility to fungal co‐infections.^6^, ^13^, ^15^

Based on past studies, cutaneous mucormycosis as the third most common clinical manifestation of this infection can be classified into three forms: localized, deep extension, and disseminated infection.^17^ Localized cutaneous mucormycosis occurs in 32%–56% of cases and is restricted to the cutaneous and subcutaneous tissues.^18^ According to previous studies, the most common risk factor for cutaneous mucormycosis in immunocompetent patients is inoculation through trauma sites (23%–88%), specifically 3%–33% of car accidents.^18^ Furthermore, wound dressing has been identified in some cases as a source of contamination that leads to cutaneous mucormycosis.^19^ Ingram et al. published a case series of cutaneous mucormycosis over a 15‐year period that was associated with vehicle accidents.^20^

In this patient, after COVID‐19 involvement, he complained of facial pain and on the physical examination, there were edema and pain in the right half of the face and head. In this case, the initial symptoms of facial mucormycosis included fever, cellulitis and vesicles, which quickly progressed to skin necrosis and black scar formation. While radiological imaging techniques was useful for evaluating the disease extent and planning the surgical approach. Because the causative fungal agent is difficult to culture, tissue biopsy is critical for diagnosis of cutaneous mucormycosis.^21^, ^22^ Mucormycosis has a high mortality rate (up to 85%) that is associated with late or no diagnosis. The patient's prognosis is principally dependent on rapid diagnosis and treatment.^23^

Our case highlights that COVID‐19 can be present with pleural effusion detection in CT. Therefore, it must be kept in mind that pleural effusion is considered as a rare CT manifestation of COVID‐19 infections.^24^ With the obvious clinical signs and symptoms of COVID‐19 infections, we considered the patient to be a COVID‐19 patient that was treated with the COVID‐19 protocols. In this global crisis, uncommon cases COVID‐19 infection diagnosis is important for reducing the transmission level and control of the current pandemic. On the other hand, if the infection is not quickly recognized, its progression is fast and fatal.^1^

At the time of hospitalization of this patient due to a car accident, the whole world and our country were suffering from a COVID‐19 pandemic, and also, the vaccination rate in our country had not yet reached an acceptable level, so that only the medical staff and high‐risk patients were vaccinated. The possibility of infection with COVID‐19 during hospitalization was very high. On the other hand, this patient has undergone two major surgeries, and thus, the level of the patient's immune system has been weakened, which is also a possible factor for being infected with COVID‐19. According to our findings, the main risk factors for facial mucormycosis include typical protective barrier disruption during a car accident and COVID‐19 involvement. The causative agent of cutaneous mucormycosis is capable of rapidly penetrating damaged tissues. Because mucormycosis is a medical emergency condition, waiting for culture results is not practical and may lead to patient death. Hence, a positive direct smear may be enough to initiate treatment protocols.^25^ Besides, Figure 2 clearly shows that the black necrotic scar surrounded by erythema is a typical sign of cutaneous mucormycosis. Finally, these observations and findings suggest a strong link between steroid therapy and mucormycosis in patients with COVID‐19 infection.

According to our case and past studies, the successful cutaneous mucormycosis therapeutic approach can be summarized into four stages.^25^, ^26^

First, patients with a high degree of suspicion require an early diagnosis. Fortunately, the current patient's diagnosis was made immediately.

Second, prompt medical and surgical therapy is required to reduce higher death rates. When treatment is started during the first 5 days, the mortality rate is significantly reduced. Lanternier et al. discovered that the median time between the onset of symptoms and diagnosis in individuals with cutaneous mucormycosis was much shorter than in patients with other clinical manifestations of mucormycosis infections.^27^

Amphotericin B is the primary antifungal treatment for mucormycosis. In our case, we initiated treatment with intravenous liposomal AMB and ITR as an oral antifungal agent has been used after a successful response to AMB to avoid AMB side effects.

Third, controlling predisposing conditions considerably improves survival rates. In our situation, we altered the use of immunosuppressive medications while simultaneously controlling predisposing factors.

Finally, surgical debridement of the infected area is almost vital to eliminate the link between angioinvasion and necrosis and reconstruction of damaged tissue in cutaneous mucormycosis.

In the current patient, surgical treatment was performed at distinct intervals and continued until a healthy margin of tissue appeared. In this study, the elements that contributed to the patient's survival included effective medical care and periodical debridement surgery.

The present case survived with proper medical therapy and regular debridement surgery. It is noteworthy that surgical debridement is so critical for cutaneous mucormycosis control and preventing rhinocerebral mucormycosis.^26^ The value of medical antifungal therapy alone is diminished by the extensive vascular tissue invasion and necrosis that limit drug penetration involved sites. Further investigations are essential for better prevention and management of opportunistic fungal infections in COVID‐19 patients. Besides, antifungal prophylaxis guidelines may need to be established and created to reduce the morbidity and mortality rates of this coinfection.

In this study, since the patient had many problems and underwent surgery twice, which caused long hospitalization in the COVID‐19 pandemic, he had post COVID‐19 mucormycosis. Probably, health care leaders should employ some implementation to facilitate safely decreasing ICU utilization, accelerating discharge for reducing the possibility of COVID‐19 transmission.

In the wake of the COVID‐19 pandemic, it is crucial to expand laboratory‐testing capacities, improve epidemiological surveillance with robust technologies invest, and institute awareness and preventive measures to progress the burden on our health services.

## 4 CONCLUSION

Mucormycosis is a potentially fatal, angioinvasive fungal infection that primarily affects patients with immunodeficiency and diabetic ketoacidosis. We present a complicated case of cutaneous mucormycosis in a COVID‐19 patient who was hospitalized following a car accident. The main pillars of this mucormycosis type treatment are surgical debridement of necrotic tissue, followed by systemic antifungal drug, and also alteration of any cause of immunosuppressive elements.

## AUTHOR CONTRIBUTIONS

Zahra Zareshahrabadi: Conceptualization (lead); writing original draft (lead); writing review and editing (equal). Keyvan Pakshir: Methodology (equal), writing review and editing (equal). Amir Emami: Methodology (lead); writing review and editing (equal). Amir Roudgari: Investigation writing original draft (supporting); writing review and editing (equal). Behzad Ghaffari: Investigation (equal); writing original draft (Equal). Tahere Rezaei: Investigation (equal); writing review and editing (equal). Golsa Shekarkhar: Methodology (equal); writing original article. Kamiar Zomorodian: Project administration (lead); supervision (lead); writing review and editing (equal).

## Funding information

No funding was received for this work.

## CONFLICT OF INTEREST

The authors report no conflicts of interest. The authors alone are responsible for the content and writing.

## ETHICAL APPROVAL

The study was approved by the ethical committee of Shiraz University of Medical Sciences, Shiraz, Iran. Informed consent was obtained from the patient prior to being included in the study.

## CONSENT

Written informed consent was obtained from the patient to publish this report in accordance with the journal‘s patient consent policy.

## Data Availability

The data used to support the findings of this study were supplied by Vice‐Chancellor for Research of Shiraz University of Medical Sciences under license. Requests for data access should be made to Kamiar Zomorodian, kzomorodian@gmail.com.
